# Factors influencing plagiarism in higher education: A comparison of German and Slovene students

**DOI:** 10.1371/journal.pone.0202252

**Published:** 2018-08-10

**Authors:** Eva Jereb, Matjaž Perc, Barbara Lämmlein, Janja Jerebic, Marko Urh, Iztok Podbregar, Polona Šprajc

**Affiliations:** 1 Department of Personnel and Education, Faculty of Organizational Sciences, University of Maribor, Kranj, Slovenia; 2 Faculty of Natural Sciences and Mathematics, University of Maribor, Maribor, Slovenia; School of Electronic and Information Engineering, Beihang University, Beijing, China; 3 Department of Economics and Law, Frankfurt University of Applied Sciences, Frankfurt, Germany; 4 Department of Methodology, Faculty of Organizational Sciences, University of Maribor, Kranj, Slovenia; Medizinische Universitat Graz, AUSTRIA

## Abstract

Over the past decades, plagiarism has been classified as a multi-layer phenomenon of dishonesty that occurs in higher education. A number of research papers have identified a host of factors such as gender, socialisation, efficiency gain, motivation for study, methodological uncertainties or easy access to electronic information via the Internet and new technologies, as reasons driving plagiarism. The paper at hand examines whether such factors are still effective and if there are any differences between German and Slovene students’ factors influencing plagiarism. A quantitative paper-and-pencil survey was carried out in Germany and Slovenia in 2017/2018 academic year, with a sample of 485 students from higher education institutions. The major findings of this research reveal that easy access to information-communication technologies and the Web is the main reason driving plagiarism. In that regard, there are no significant differences between German and Slovene students in terms of personal factors such as gender, motivation for study, and socialisation. In this sense, digitalisation and the Web outrank national borders.

## Introduction

Many of those who teach in higher education have encountered the phenomenon of plagiarism as a form of dishonesty in the classroom. According to the Oxford English Dictionary online 2017, the term plagiarism is defined as ‘the practice of taking someone else's work or ideas and passing them off as one's own’. Perrin, Larkham and Culwin define plagiarism as the use of an author's words, ideas, reflections and thoughts without proper acknowledgment of the author [1–3]. Koul et al. define plagiarism as a form of cheating and theft since in cases of plagiarism one person takes credit for another person’s intellectual work [4]. According to Fishman, ‘Plagiarism occurs when someone: 1) uses words, ideas, or work products; 2) attributable to another identifiable person or source; 3) without attributing the work to the source from which it was obtained; 4) in a situation in which there is a legitimate expectation of original authorship; 5) in order to obtain some benefit, credit, or gain which need not be monetary’ [5]. But why do students use someone else's words or ideas and pass them on as their own? Which factors influence this behaviour? That is the main focus of our research, to discover the factors influencing plagiarism and see if there are any differences between German and Slovene students.

Koul et al. pointed out that particular circumstances or events should be considered in the definition of plagiarism since plagiarism may vary across cultures and societies [4]. Hall has described Eastern cultures (the Middle East, Asia, Africa, South America) and Western cultures (North America and much of Europe) using the idea of ‘context’, which refers to the framework, background, and surrounding circumstances in which an event takes place [6]. Western societies are generally ‘low context’ societies. In other words, people in Western societies play by external rules (e.g., honour codes against plagiarism), and decisions are based on logic, facts, and directness. Eastern societies are generally ‘high context’ societies, meaning that people in Eastern societies put strong emphasis on relational concerns, and decisions are based on personal relationships. Nisbett et al. have suggested that differences between Westerners and Easterners may arise from people being socialised into different worldviews, cognitive processes and habits of mind [7]. In Germany, there has been ongoing reflection on academic plagiarism and other dishonest research practices since the late 19th century [8]. However, according to Ruiperez and Garcia-Cabrero, in Germany, 2011 became a landmark year with the appearance of an extensive public debate about plagiarism—brought back into the limelight because of an investigation into the incumbent German Defence Minister’s doctoral thesis [9]. Aside from the numerous cases of plagiarism detected in academic work since 2011, several initiatives have enriched the debate on academic plagiarism. For example, the development of a consolidated cooperative textual research methodology using a specific Wiki called ‘VroniPlag’ has made Germany one of the most advanced European countries in terms of combating these practices. Similar to Germany, Slovenia has also paid increased attention to plagiarism in recent years. The debate about plagiarism became public after it was discovered that certain Slovene politicians had resorted to academic plagiarism. Today, universities in Slovenia use a variety of tools (Turnitin, plagiarism plug-ins for Moodle, plagiarisma.net, etc.) in order to detect plagiarism. The focus of this research is to investigate the factors influencing plagiarism and if there are any differences between Slovene and German students’ factors influencing plagiarising. The research questions (RQ) of the study were divided into three groups:

- RQ group 1: Which factors influence plagiarism in higher education?
- RQ group 2: Are there any differences between male and female students regarding factors influencing plagiarism? Are the factors influencing plagiarism connected with specific areas of study (technical sciences, social sciences, natural sciences)?
- RQ group 3: Does the students’ motivation affect their factors influencing plagiarism? Are there any differences between male and female students regarding this?

In addition, for all three research question groups, we also wanted to know if there were any differences between the German and Slovene students.

### Theoretical background

Plagiarism is a highly complex phenomenon and, as such, it is likely that there is no single explanation for why individuals engage in plagiarist behaviours [10]. The situation is often complex and multi-dimensional, with no simple cause-and-effect link [11].

McCabe et al. noted that individual factors (e.g. gender, average grade, work ethic, self-esteem), institutional factors (e.g., faculty response to cheating, sanction threats, honour codes) and contextual factors (e.g., peer cheating behaviours, peer disapproval of cheating behaviours, perceived severity of penalties for cheating) influence cheating behaviour [12]. Giluk and Postlethwaite also related individual characteristics and situational factors to cheating—individual characteristics such as gender, age, ability, personality, and extracurricular involvement; and situational factors such as honour codes, penalties, and risk of detection [13]. The study of Jereb et al. also revealed that specific individual characteristics pertaining to men and women influence plagiarism [14]. Newstead et al. suggested that gender differences (plagiarism is more frequent among boys), age differences (plagiarism is more frequent among younger students), and academic performance differences (plagiarism is more frequent among lower performers) are specific factors for plagiarism [15]. Gerdeman stated that the following five student characteristic variables are frequently related to the incidence of dishonest behaviour: academic achievement, age, social activities, study major, and gender [16].

One of the factors influencing plagiarism could be that students do not have a clear understanding of what constitutes plagiarism and how it can be avoided [17, 18]. According to Hansen, students don’t fully understand what constitutes plagiarism [19]. Park states genuine lack of understanding as one of the reasons for plagiarism. Some students plagiarise unintentionally, when they are not familiar with proper ways of quoting, paraphrasing, citing and referencing and/or when they are unclear about the meaning of ‘common knowledge’ and the expression ‘in their own words’ [11].

Furthermore, it is important to remember that, in our current day and age, information is easily accessed through new technologies. In addition, as Koul et al. have stated, the belief that we as people have greater ownership of information than we have paid for may influence attitudes towards plagiarism [4]. Many other authors have also stated that the Internet has increased the potential for plagiarism, since information is easily accessed through new technologies [14, 20, 21, 22]. Indeed, the Internet grants easy access to an enormous amount of knowledge and learning materials. This provides an opportunity for students to easily cut, paste, download and plagiarise information [21, 23]. Online resources are available 24/7 and enable a flood of information, which is also constantly updated. Given students' ease of access to both digital information and sophisticated digital technologies, several researchers have noted that students may be more likely to ignore academic ethics and to engage in plagiarism than would otherwise be the case [24].

In a study of the level of plagiarism in higher education, Tayraukham found that students with performance goals were more likely to indulge in plagiarism behaviours than students who wanted to achieve mastery of a particular subject [25]. Most of the students plagiarised in order to provide the right responses to study questions, with the ultimate goal of getting higher grades—rather than gaining expertise in their subjects of study. Anderman and Midgley observed that a relatively higher performance-oriented classroom climate increases cheating behaviour; while a higher mastery-oriented classroom climate decreases cheating behaviour [26]. Park also claimed that one of the reasons that students plagiarise is efficiency gain, that is, that students plagiarise in order to get a better grade and save time [11]. Songsriwittaya et al. stated that what motivates students to plagiarise is the goal of getting good grades and comparing their success with that of their peers [27]. The study of Ramzan et al. also revealed that the societal and family pressures of getting higher grades influence plagiarism [21]. Such pressures sometimes push students to indulge in unfair means such as plagiarism as a shortcut to performing better in exams or producing a certain number of publications. Engler et al. and Hard et al. tended to agree with this idea, stating that plagiarism arises out of social norms and peer relationships [28, 29]. Park also stated that there are many calls on students’ time, including peer pressure for maintaining an active social life, commitment to college sports and performance activities, family responsibilities, and pressure to complete multiple work assignments in short amounts of time [11]. Šprajc et al. agreed that students are under an enormous amount of pressure from family, peers, and instructors, to compete for scholarships, admissions, and, of course, places in the job market [30]. This affects students’ time management and can lead to plagiarism. In addition to time pressures, Franklin-Stokes and Newstead found another six major reasons given by students to explain cheating behaviours: the desire to help a friend, a fear of failure, laziness, extenuating circumstances, the possibility of reaping a monetary reward, and because ‘everybody does it’ [31].

Another common reason for plagiarism is the poor preparation of lecture notes, which can lead to the inadequate referencing of texts [32]. Šprajc et al. found out that too many assignments given within a short time frame pushes students to plagiarise [30]. Poor explanations, bad teaching, and dissatisfaction with course content can also drive students to plagiarise. Park exposed students’ attitudes towards teachers and classes [11]. Some students cheat because they have negative attitudes towards assignments and tasks that teachers believe to have meaning but that they don’t [33]. Cheating tends to be more common in classes where the subject matter seems unimportant or uninteresting to students, or where the teacher seemed disinterested or permissive [16].

Park mentioned students’ academic skills (researching and writing skills, knowing how to cite, etc.) as another reason for plagiarism [11]. New students and international students whose first language is not English need to transition to the research culture by understanding the necessity of doing research, and the practice and skills required to do so, in order to avoid unintentional plagiarism [21]. According to Park to some students, plagiarism is a tangible way of showing dissent and expressing a lack of respect for authority [11]. Some students deny to themselves that they are cheating or find ways of legitimising their behaviour by passing the blame on to others. Other factors influencing plagiarising are temptation and opportunity. It is both easier and more tempting for students to plagiarise since information has become readily accessible with the Internet and Web search tools, making it faster and easier to find information and copy it. In addition, some people believe that since the Internet is free for all and a public domain, copying from the Internet requires no citation or acknowledgement of the source [34]. To some students, the benefits of plagiarising outweigh the risks, particularly if they think there is little or no chance of getting caught and there is little or no punishment if they are indeed caught [35].

One of the factors influencing plagiarism could be also higher institutions’ attitudes towards plagiarism, that is, whether they have clear policies regarding plagiarism and its consequences or not. The effective communication of policies, increased student awareness of penalties, and enforcement of these penalties tend to reduce dishonest behaviour [36]. Ramzan et al. [21] mentioned the research of Razera et al., who found that Swedish students and teachers need training to understand and avoid plagiarism [37]. In order to deal with plagiarism, teachers want and need a clear set of policies regarding detection tools, and extensive training in the use of detection software and systems. According to Ramzan et al., Dawson and Overfield determined that students are aware that plagiarism is bad but that they are not clear on what constitutes plagiarism and how to avoid it [21, 38]. In Dawson and Overfield’s study, students required teachers to also observe the rules set up to avoid plagiarism and be consistently kept aware of plagiarism—in order to enforce the university’s resolve to control this academic misconduct.

According to this literature review and our experiences in higher education teaching, we determined that the following factors influence plagiarism: students’ individual factors, information-communication technologies (ICT) and the Web, regulation, students’ academic skills, teaching factors, different forms of pressure, student pride, and other reasons. The statements used in the instrument we developed, and the results of our research are presented in the following chapters.

## Method

### Participants

The paper-and-pencil survey was carried out in the 2017/18 academic year at the University of Maribor in Slovenia and at the Frankfurt University of Applied Sciences in Germany. Students were verbally informed of the nature of the research and invited to freely participate. They were assured of anonymity. The study was approved by the Ethical Committee for Research in Organizational Sciences at Faculty of Organizational Sciences University of Maribor.

A sample of 191 students from Slovenia (SLO) (99 males (51.8%) and 92 (48.2%) females) and 294 students from Germany (GER) (115 males (39.1%) and 171 (58.2%) females) participated in this study. Slovene students’ ages ranged from 19 to 36 years, with a mean of 21 years and 1 months (*M = 21*.*12* and *SD = 1*.*770*) and German students’ ages ranged from 18 to 40 years, with a mean of 22 years and 10 months (*M = 22*.*84* and *SD = 3*.*406*). About half (49.2%) of the Slovene participants were social sciences students, 34.9% were technical sciences students, and 15.9% were natural sciences students. More than half (58.5%) of the German participants were social sciences students, 32% were technical sciences students and 2% were natural sciences students. More than half of the Slovene students (53.4%) attended blended learning, and 46.6% attended classic learning. The majority of German students (87.8%) attended classic learning, and 6.8% attended blended learning. More than half of the Slovene students (61.6%) were working at the time of the study, and 39.8% of all participants had scholarships. In addition, in Germany, more than half the students (65.0%) were working at the time of the study, but only 10.2% of all the German participants had scholarships. More than two thirds (68.9%) of the Slovene students were highly motivated for study and 31.1% less so; 32.6% of the students spend 2 or fewer hours per day on the Internet, 41.6% spend between 2 and 5 hours on the Internet, and 25.8% spend 5 or more hours on the Internet per day. Also, more than two thirds (73.1%) of the German students were highly motivated for study and 23.8% less so; 33.3% of the students spend 2 or fewer hours per day on the Internet, 32.3% spend between 2 and 5 hours on the Internet, and 27.9% spend 5 or more hours on the Internet per day. The general data can be seen in S1 Table.

### Instrument

The questionnaire contained closed questions referring to: (i) general/individual data (gender, age, area of study, method of study, working status, scholarship, motivation for study, average time spent on the internet), and factors influencing plagiarism (ii) ICT and Web, (iii) regulation, (iv) academic skills, (v) teaching factors, (vi) pressure, (vii) pride, (viii) other reasons. The items in the groups (ii) to (viii) used a 5-point Likert scale from strongly disagree (1) to strongly agree (5), with larger values indicating stronger orientation.

The statements used in the survey were as follows:

1 ICT and Web
  1.1 It is easy for me to copy/paste due to contemporary technology
  1.2 I do not know how to cite electronic information
  1.3 It is hard for me to keep track of information sources on the web
  1.4 I can easily access research material using the Internet
  1.5 Easy access to new technologies
  1.6 I can easily translate information from other languages
  1.7 I can easily combine information from multiple sources
  1.8 It is easy to share documents, information, data
**2 Regulation**
  2.1 There is no teacher control on plagiarism
  2.2 There is no faculty regulation against plagiarism
  2.3 There is no university regulation against plagiarism
  2.4 There are no penalties
  2.5 There are no honour codes relating to plagiarism
  2.6 There are no electronic systems of control
  2.7 There is no systematic tracking of violators
  2.8 I will not get caught
  2.9 I am not aware of penalties
  2.10 I do not understand the consequences
  2.11 The penalties are minor
  2.12 The gains are higher than the losses
**3 Academic skills**
  3.1 I run out of time
  3.2 I am unable to cope with the workload
  3.3 I do not know how to cite
  3.4 I do not know how to find research materials
  3.5 I do not know how to research
  3.6 My reading comprehension skills are weak
  3.7 My writing skills are weak
  3.8 I sometimes have difficulty expressing my own ideas
**4 Teaching factors**
  4.1 The tasks are too difficult
  4.2 Poor explanation—bad teaching
  4.3 Too many assignments in a short amount of time
  4.4 Plagiarism is not explained
  4.5 I am not satisfied with course content
  4.6 Teachers do not care
  4.7 Teachers do not read students' assignments
**5 Pressure**
  5.1 Family pressure
  5.2 Peer pressure
  5.3 Under stress
  5.4 Faculty pressure
  5.5 Money pressure
  5.6 Afraid to fail
  5.7 Job pressure
**6 Pride**
  6.1 I do not want to look stupid in front of peers
  6.2 I do not want to look stupid in front of my professor
  6.3 I do not want to embarrass my family
  6.4 I do not want to embarrass myself
  6.5 I focus on how my competences will be judged relative to others
  6.6 I am focused on learning according to self-set standards
  6.7 I fear asking for help
  6.8 My fear of performing poorly motivates me to plagiarise
  6.9 Assigned academic work will not help me personally/professionally
**7 Other factors**
  7.1 I do not want to work hard
  7.2 I do not want to learn anything, just pass
  7.3 My work is not good enough
  7.4 It is easier to plagiarise than to work
  7.5 To get a better/higher mark (score)

## Results

All statistical tests were performed with SPSS at the significance level of 0.05. Parametric tests (Independent–Samples t-Test and One-Way ANOVA) were selected for normal and near-normal distributions of the responses. Nonparametric tests (Mann-Whitney Test, Kruskal-Wallis Test, Friedman’s ANOVA) were used for significantly non-normal distributions. Chi-Square Test was used to investigate the independence between variables.

### RQ group 1

The average values for the groups (and standard deviations) of the responses referring to the factors influencing plagiarism can be seen in Table 1 (descriptive statistics for all statements can be seen in S2 Table), shown separately for Slovene and German students. An Independent Samples t-test was conducted to obtain the average values of the responses, and thus evaluate for which statements these differed significantly between the Slovene and German students.

**Table 1:** Descriptive statistics for groups referring to the factors influencing plagiarism, by country and results of the t-Test.

	Factors influencing plagiarism	SLO		GER		t-Test	
		*M*	*SD*	*M*	*SD*	*t*	*p*
1	ICT and Web	3.69	0.56	3.47	0.55	4.177	**
2	Regulation	2.35	0.63	2.05	0.61	5.137	**
3	Academic skills	2.56	0.67	2.44	0.68	1.939	
4	Teaching factors	2.87	0.68	2.56	0.72	4.827	**
5	Pressure	2.42	0.86	2.71	0.91	-3.522	**
6	Pride	2.43	0.84	2.67	0.80	-3.032	**
7	Other reasons	2.47	0.82	2.54	0.94	-0.836	

Note.

*p < .05.

**p < .01

According to the Friedman’s ANOVA (see Table 2), the Slovene students’ factors influencing plagiarism can be formed into four homogeneous subsets, where in each subset, the distributions of the average values for the responses are not significantly different. At the top of the list is the existence of ICT and the Web (group 1). The second subset consists of teaching factors (group 4). The third subset is composed of academic skills, other reasons, and pride, in order from highest to lowest (groups 3, 7 and 6). The fourth subset is composed of other reasons, pride, pressure, and regulation, respectively (groups 7, 6, 5 and 2).

**Table 2:** Homogeneous subsets by Friedman’s ANOVA for Slovene students.

	Factors influencing plagiarism	Sample average rank			
		Subset 4	Subset 3	Subset 2	Subset 1
2	Regulation	3.097			
5	Pressure	3.204			
6	Pride	3.369	3.369		
7	Other reasons	3.490	3.490		
3	Academic skills		3.654		
4	Teaching factors			4.738	
1	ICT and Web				6.448
	Test Statistic	5.458	3.097		
	Sig (2-sided)	0.141	0.213		

For the Slovene students, ICT and the Web were detected as the dominant factors influencing plagiarism and, as such, we investigated them in greater detail. A Friedman Test (*Chi-Square =* 7.180, *p* = .066) confirmed that the distributions of the responses to the statements 1.1, 1.4, 1.5 and 1.8—those with the highest sample means—are not significantly different. Consequently, the average values (means) of the responses to the statements 1.1, 1.4, 1.5 and 1.8 are not significantly different. The average values of the responses for all the other statements (1.7, 1.6, 1.2, and 1.3 listed in the descending order of sample means) are significantly lower. A Mann-Whitney Test showed that there is no statistically significant difference between the distributions of the responses in the group of ICT and Web reasons considering gender (male, female) and motivation for study (lower, higher). For statement 1.2, a Kruskal-Wallis Test (*Chi-Square =* 7.466, *p =* .024) confirmed that there are different distributions for the responses when the area of study is considered (technical sciences, social sciences, natural sciences).

According to the Friedman’s ANOVA (see Table 3), the German students’ factors influencing plagiarism can be formed into five homogeneous subsets, where in each subset, the distributions of the average values for the responses are not significantly different. At the top of the list is the existence of ICT and the Web (group 1). The second subset is composed of pressure and pride, in order from highest to lowest (groups 5 and 6). The third subset consists of pride, teaching factors and other reasons, respectively (groups 6, 4 and 7). The fourth subset is composed of teaching factors, other reasons and academic skills, in order from highest to lowest (groups 4, 7 and 3). Finally, the last subset consists of regulation (group 2).

**Table 3:** Homogeneous subsets by Friedman’s ANOVA for German students.

	Factors influencing plagiarism	Sample average rank				
		Subset 5	Subset 4	Subset 3	Subset 2	Subset 1
2	Regulation	2.465				
3	Academic skills		3.392			
7	Other reasons		3.745	3.745		
4	Teaching factors		3.799	3.799		
6	Pride			4.132	4.132	
5	Pressure				4.368	
1	ICT and Web					6.099
	Test Statistic		7.578	7.651	4.048	
	Sig (2-sided)		0.052	0.05	0.146	

Just like the Slovene students, for the German students ICT and the Web were detected as the dominant factors influencing plagiarism. That the distributions of the responses to the statements 1.4, 1.5 and 1.8—those with the highest sample means—are not significantly different was confirmed by Friedman Test (*Chi-Square =* 5.815, *p =* .055). Consequently, the average values (means) of the responses to the statements 1.4, 1.5 and 1.8 are not significantly different. The average values of the responses for all the other statements (1.1, 1.7, 1.6, 1.2, and 1.3 listed in the descending order of sample means) are significantly lower. A Wilcoxon Signed Ranks Tests also confirmed that the distributions of the responses to the statements 1.6 and 1.7 are not statistically significantly different (*Z =* -0.430, *p =* .667). The same holds for statements 1.2 and 1.3 (*Z =* -0.407, *p =* .684). A Mann-Whitney Test showed that there is no statistically significant difference between the distributions of the responses in the group of ICT and Web reasons considering gender (male, female), area of study (technical and social sciences (students of natural sciences were omitted due to the small sample size)) and motivation for study.

ICT and Web reasons were detected as the dominant factors influencing plagiarism for Slovene and German students. As can be seen in Table 1, there are significant differences (*t =* 4.177, *p =* .000*)* between the Slovene and German students regarding this factor. It seems that the Slovene students (*M =* 3.69, *SD =* 0.56) attribute greater importance to the ICT and Web reasons than the German students (*M =* 3.47, *SD =* 0.55). There are also significant differences (*t =* 5.137, *p =* .000*)* between the Slovene and German students regarding regulation. It seems that the Slovene students (*M =* 2.35, *SD =* 0.63) attribute greater importance to regulation reasons than the German students (*M =* 2.05, *SD =* 0.61). Both, however, consider this factor to have the lowest impact on plagiarism overall. There are no significant differences (*t =* 1.939, *p =* .053*)* between the Slovene students (*M =* 2.56, *SD =* 0.67) and the German students (*M =* 2.44, *SD =* 0.68) regarding academic skills. The Slovene students (*M =* 2.87, *SD =* 0.68) attribute greater importance to teaching factors than the German students (*M =* 2.56, *SD =* 0.72). The differences are significant (*t =* 4.827, *p =* .000*)*. There are significant differences (*t =* -3.522, *p =* .000*)* between the Slovene and German students regarding pressure, whereas the German students (*M =* 2.71, *SD =* 0.91) attribute greater importance to this reason than the Slovene students (*M =* 2.42, *SD =* 0.86). The same goes for pride. The German students (*M =* 2.67, *SD =* 0.80) attribute greater importance to pride reasons than the Slovene students (*M =* 2.43, *SD =* 0.84). The differences are significant (*t =* -3.032, *p =* .003*)*. There are no significant differences (*t = -*0.836, *p =* .404*)* between the Slovene students (*M =* 2.47, *SD =* 0.82) and the German students (*M =* 2.54, *SD =* 0.94) regarding other factors influencing plagiarism.

We conducted an Independent Samples t-test to compare the average time (in hours) spent per day on the Internet by the Slovene students with that of the German students. The test was significant, *t =* -2.064, *p* = .004. The Slovene students on average spent less time on the Internet (*M =* 3.52, *SD =* 2.23) than the German students (*M =* 4.09, *SD =* 3.72).

### RQ group 2

The average values of the responses for individual statements according to gender (male, female) and the significances for the t-test of equality of means are shown in S3 Table for the Slovene students and in S4 Table for the German students. The average values of the responses for these statements are significantly different. They are higher for males than for females (except in the case of statement 3.8 for the Slovene students and 4.1 for the German students). Slovene and German male students think that they will not get caught and that the gains are higher than the losses. Both also think that teachers do not read students’ assignments.

The average values of the responses for individual statements according to area of study (technical sciences, social sciences, natural sciences) and the results for ANOVA for the Slovene students are shown in S5 Table. Gabriel's post hoc test was used to confirm the differences between groups. The significant difference between the students of technical sciences and the students of social sciences was confirmed for all statements listed in S5 Table. There were higher average values of responses for the students of technical sciences. The only significant difference between the students of technical sciences and the students of natural sciences was confirmed for statement 5.6 (there were higher average values of responses for the students of technical sciences). No other pairs of group means were significantly different.

The average values of the responses for individual statements according to area of study (technical sciences, social sciences) and the significances for the t-test of equality of means for German students are shown in S6 Table. For German students, only technical and social sciences were considered because of the low number of natural sciences students. The average values of responses for these statements are significantly different. They were higher for the students of technical sciences than for the students of social sciences.

### RQ group 3

The average values of the responses for individual statements according to the motivation of the students (lower, higher) and the significances for t-Test of equality of means are shown in S7 Table for the Slovene students and in S8 Table for the German students. The average values of the responses for these statements are significantly different. They were higher for students with lower motivation for both groups of students, except in the case of statements 2.1 and 6.6 for Slovene students.

We conducted an Independent Samples t-test to compare the average time (in hours) spent per day on the Internet by groups of low motivated students with groups of highly motivated students. For Slovene students, the test was not significant, *t =* -1.423, *p =* .156. For German students, the test was significant, *t =* 2.298, *p =* .024. Students with lower motivation for study (*M =* 5.24, *SD =* 4.84) on average spent more time on the Internet than those with higher motivation for study (*M =* 3.76, *SD =* 3.27).

The Chi-Square Test of Independence was used to determine whether there is an association between gender (male, female) and motivation for study (lower, higher). There was a significant association between gender and motivation for the Slovene students (*Chi-Square =* 4.499, *p =* .034). Indeed, it was more likely for females to have a high motivation for study (76.9%) than for males to have a high motivation for study (61.6%). For the German students, the test was not significant (*Chi-Square =* 0.731, *p =* .393).

## Discussion

In this study, we aimed to explore factors that influence students’ factors influencing plagiarism. An international comparison between German and Slovene students was made. Our research draws on students from two universities from the two considered countries that cover all traditional subjects of study. In this regard the conclusions are representative and statistically relevant, although we of course cannot exclude the possibility of small deviations if other or more institutions would be considered. Taken as a whole, there are no major differences between German and Slovene students when it comes to motivation for study and working habits. In both cases, more than two thirds of the students were highly motivated for study and more than 60% were working during their time of study. About 33% of the surveyed students spend on average two or less hours a day on the Internet, and about one quarter spend on average more than five hours a day on the Internet.

When it comes to explaining plagiarism in higher education, the German and Slovene students equally indicated the ease-of-use of information-communication technologies and the Web as the top one cause for their behaviour. Which does not lag behind other notions of current contributions to the topic of plagiarism in the world. Indeed, our findings reinforce the notion that new technologies and the Web have a strong influence on students and are the main driver behind plagiarism [20, 21, 22]. An academic moral panic has been caused by the arrival in higher education of a new generation of younger students [39], deemed to be ‘digital natives’ [40] and allegedly endowed with an inherent ability for using information-communication technologies (ICT). This younger generation is dubbed ‘Generation Me’ [41], and it is believed that their expectations, interactions and learning processes have been affected by ICT. Introna, et al., Ma et al., and Yeo, agree that the understanding of the concept of plagiarism through the use of ICT is the main contributor to it being a problem [42, 43, 44]. The effortless use of ICT such as the Internet has made it easy for students to retrieve information with a simple click of the mouse [45, 46].

The Slovene students in our study nominated the teaching factor as the second most important reason for plagiarism. This result is also found in other studies, namely those of Šprajc et al. [30] and Barnas [47]. Young people in Slovenia are, like in other Western societies, given a prolonged period of identity exploration and self-focus, i.e., freedom from institutional demands and obligations, competence, and freedom to decide for themselves [48, 49]. The results of the German students however, contradict this finding that teaching factors are one of the most important factors influencing plagiarism. Indeed, the top two factors influencing plagiarism for the German students are actually pressure and pride—and not teaching factors. Overall though, the findings for both the German students and the Slovene students are in line with e.g. Koul et al., who suggest that factors influencing plagiarism may vary across cultures [4]. Among German students, the pressure and pride in the second and third places in terms of importance are mostly reflected, which does not lag behind the mention of the author Rothenberg stated that in Germany today ‘pride could be expressed for individual accomplishments’ [50]. As far as the Slovene students are concerned, the authors Kondrič et al. presumed that there is a specific set of values in Slovenia, which perhaps intensify the distinction between the collectivist culture of former socialist countries and the individualism of Western countries [51]. This might shed light on why the Slovene students consider teaching factors as being one of the most important factors influencing plagiarism.

Furthermore, several studies have implied that individual characteristics, especially gender, play an important role when it comes to plagiarism [12, 13, 15, 16]. A number of studies from around the world have shown that men more frequently plagiarise than women do. For example, Reviews of North American’s research into conventional plagiarism has indicated that male students cheat more often than female students [12]. The results we found are basically in line with these findings. Since the average values of responses are significantly different for male and female students, gender seems to play an important role in terms of plagiarism.

Park pointed out that one reason for plagiarism is efficiency gain [11]. About 15 years after this statement, the study at hand is empirical evidence that efficiency gain due to different forms of pressure is still a factor that influences students’ behaviour in terms of plagiarism. Lack of knowledge and uncertainties about methodologies are additional factors that are frequently recognized as reasons for plagiarism [11, 17, 18]. The results at hand support these studies since the responses about e.g. academic skills demonstrate students’ lack of knowledge.

Another interesting finding of our study shows that students with a lower motivation for study spend more time on the Internet, which complements our finding that the Internet is one of the simplest solutions for studying. The German students showed a somewhat higher level of motivation to study than the Slovene students, but the difference is not statistically significant.

We would nevertheless like to draw attention to the perceived difference, which refers to the perception of the factors influencing the plagiarism of the teacher factors and academic skills (Slovene students) and pride and pressure (German students). The perceived difference between students is one of the social dimensions that represents a tool to promote true motivation for study and proper orientation without ethically disputable solutions (such as plagiarism). In all this, it makes sense to direct students and educate them from the beginning of education together with information technology, while also builds responsible individuals who will not take technology and the Internet as a negative tool for studying and succeeding, but to help them to solve and make decisions in the right way. The main aim of this research into Slovene and German students was to increase understanding of students’ attitudes towards plagiarism and, above all, to identify the reasons that lead students to plagiarise. On this basis, we want to expose the way of non-plagiarism promotion to be developed in a way that will be more acceptable and more understandable in each country and adequately controlled on a personal and institutional level.

## Conclusions

In contrast to a number of preliminary studies, the major findings of this research paper indicate that new technologies and the Web have a strong and significant influence on plagiarism, whereas in this specific context gender and socialisation factors do not play a significant role. Since the majority of the students in our study believe that new technologies and the Web have a strong influence on plagiarism, we can assume that technological progress and globalisation has started breaking down national frontiers and crossing cultural boundaries. These findings have also created the impression that at universities the gender gap is not predominant in all areas as it might be in society.

Nevertheless, some minor results in our study indicate that there are still some differences between Slovene and German students. For example, it seems like in Slovenia, teaching factors have a greater influence on plagiarism than in Germany. Indeed, in Germany, the focus should rest on the implementation and publication of a code of ethics, and on training students to deal with pressure.

This research focuses on only two countries, Slovenia and Germany. Thus, the findings at hand are not necessarily generalizable, though they do manifest a certain trend in terms of the reasons why students resort to plagiarism. Furthermore, the results could be a starting point for additional comparative studies between different European regions. In particular, further research into the influence of digitalization and the Web on plagiarism, and the role of socialisation and gender factors on plagiarism, could contribute to the discourse on plagiarism in higher education institutions.

Understanding the reasons behind plagiarism and fostering awareness of the issue among students might help prevent future academic misconduct through increased support and guidance during students’ time studying at the university. In this sense, further reflection on preventive measures is required. Indeed, rather than focusing on the detection of plagiarism, focusing on preventive measures could have a positive effect on good scientific practice in the near future.

## Supporting information

S1 Table Frequency distributions of the study variables. (DOCX)

S2 Table Descriptive statistics for items referring to the factors influencing plagiarism, by nationality and results of the t-Test. (DOCX)

S3 Table Descriptive statistics for items referring to the factors influencing plagiarism, by gender and results of the t-Test (SLO). (DOCX)

S4 Table Descriptive statistics for items referring to the factors influencing plagiarism, by gender and results of the t-Test (GER). (DOCX)

S5 Table Descriptive statistics for items referring to the factors influencing plagiarism, by area of study and results of the One-Way ANOVA (SLO). (DOCX)

S6 Table Descriptive statistics for items referring to the factors influencing plagiarism, by study area and results of the t-Test (GER). (DOCX)

S7 Table Descriptive statistics for items referring to the factors influencing plagiarism, by motivation and results of the t-Test (SLO). (DOCX)

S8 Table Descriptive statistics for items referring to the factors influencing plagiarism, by motivation and results of the t-Test (GER). (DOCX)

S1 File Individual data. (XLSX)
