# Supplementary material for: Factors influencing plagiarism in higher education: A comparison of German and Slovene students
Source: PLoS One. 2018 Aug 10;13(8):e0202252. doi: 10.1371/journal.pone.0202252 (PMC6086479; doi:10.1371/journal.pone.0202252)
Supplement: S1 Table — (DOCX) [file pone.0202252.s001.docx]

**S1 Table. Frequency distributions of the study variables.**

|  |  | **SLO**  **(*n* = 191)** | | **GER**  **(*n*=294)** | |
| --- | --- | --- | --- | --- | --- |
| **Gender** | Male | 99 | 51.8% | 115 | 39.1% |
|  | Female | 92 | 48.2% | 171 | 58.2% |
|  | Missing | 0 | 0% | 8 | 2.7% |
| **Area of study** | Technical sciences | 66 | 34.9% | 94 | 32.0% |
|  | Social sciences | 93 | 49.2% | 172 | 58.5% |
|  | Natural sciences | 30 | 15.9% | 2 | 0.7% |
|  | Missing | 2 | 1.0% | 26 | 8.8% |
| **Method of study** | Classic learning | 89 | 46.6% | 258 | 87.8% |
|  | Blended learning | 102 | 53.4% | 20 | 6.8% |
|  | Missing | 0 | 0% | 16 | 5.4% |
| **Working during the time of study** | Yes | 117 | 61.6% | 191 | 65.0% |
|  | No | 73 | 38.4% | 95 | 32.3% |
|  | Missing | 1 | 0.5% | 8 | 2.7% |
| **Scholarship** | Yes | 76 | 39.8% | 30 | 10.2% |
|  | No | 115 | 60.2% | 244 | 83.0% |
|  | Missing | 0 | 0% | 20 | 6.8% |
| **Motivation for study** | Lower | 59 | 31.1% | 70 | 23.8% |
|  | Higher | 131 | 68.9% | 215 | 73.1% |
|  | Missing | 1 | 0.5% | 9 | 3.1% |
| **Average time spent on the Internet, in hours** | 2 or less hours | 62 | 32.6% | 98 | 33.3% |
|  | Between 2 and 5 hours | 79 | 41.6% | 95 | 32.3% |
|  | 5 or more hours | 49 | 25.8% | 82 | 27.9% |
|  | Missing | 1 | 0.5% | 19 | 6.5% |
