# Supplementary material for: Factors influencing plagiarism in higher education: A comparison of German and Slovene students
Source: PLoS One. 2018 Aug 10;13(8):e0202252. doi: 10.1371/journal.pone.0202252 (PMC6086479; doi:10.1371/journal.pone.0202252)
Supplement: S2 Table — (DOCX) [file pone.0202252.s002.docx]

**S2 Table. Descriptive statistics for items referring to the factors influencing plagiarism, by nationality and results of the t-Test.**

|  | **Factors influencing**  **plagiarism** | **SLO** | |  | **GER** | |  | **t-Test** | |
| --- | --- | --- | --- | --- | --- | --- | --- | --- | --- |
|  |  | ***M*** | ***SD*** |  | ***M*** | ***SD*** |  | ***t*** | ***p*** |
| 1.1 | It is easy for me to copy/paste due to contemporary technology | 4.24 | 0.83 |  | 3.78 | 1.04 |  | 5.293 | ****** |
| 1.2 | I do not know how to cite electronic information | 2.37 | 1.04 |  | 2.44 | 0.97 |  | -0.771 |  |
| 1.3 | It is hard for me to keep track of information sources on the web | 2.95 | 1.01 |  | 2.42 | 1.01 |  | 5.526 | ****** |
| 1.4 | I can easily access research material using the Internet | 4.14 | 0.80 |  | 4.03 | 0.96 |  | 1.310 |  |
| 1.5 | Easy access to new technologies | 4.17 | 0.80 |  | 3.92 | 0.97 |  | 2.863 | ****** |
| 1.6 | I can easily translate information from other languages | 3.60 | 1.09 |  | 3.56 | 0.92 |  | 0.389 |  |
| 1.7 | I can easily combine information from multiple sources | 3.88 | 0.95 |  | 3.58 | 0.82 |  | 3.613 | ****** |
| 1.8 | It is easy to share documents, information, data | 4.14 | 0.82 |  | 3.96 | 0.96 |  | 2.066 | ***** |
| **1** | **ICT and Web** | **3.69** | **0.56** |  | **3.47** | **0.55** |  | **4.177** | ****** |
|  | **Factors influencing**  **plagiarism** | **SLO** | |  | **GER** | |  | **t-Test** | |
|  |  | ***M*** | ***SD*** |  | ***M*** | ***SD*** |  | ***t*** | ***p*** |
| 2.1 | There is no teacher control on plagiarism | 2.46 | 0.88 |  | 2.02 | 0.884 |  | 5.287 | ****** |
| 2.2 | There is no faculty control on plagiarism | 2.25 | 0.83 |  | 2.00 | 0.888 |  | 3.055 | ****** |
| 2.3 | There is no university control on plagiarism | 2.17 | 0.80 |  | 1.85 | 0.881 |  | 4.095 | ****** |
| 2.4 | There are no penalties | 2.03 | 0.87 |  | 1.76 | 0.960 |  | 3.091 | ****** |
| 2.5 | There are no honour codes on plagiarism | 2.35 | 0.86 |  | 2.43 | 0.994 |  | -0.946 |  |
| 2.6 | There are no electronic systems of control | 2.13 | 0.92 |  | 2.16 | 1.064 |  | -0.313 |  |
| 2.7 | There is no systematic tracking on violators | 2.51 | 1.01 |  | 2.37 | 0.930 |  | 1.571 |  |
| 2.8 | I will not get caught | 2.14 | 1.05 |  | 2.04 | 1.018 |  | 1.077 |  |
| 2.9 | I am not aware of penalties | 2.61 | 1.13 |  | 2.06 | 1.075 |  | 5.292 | ****** |
| 2.10 | I do not understand the consequences | 2.61 | 1.12 |  | 1.81 | 1.002 |  | 7.951 | ****** |
| 2.11 | The penalties are minor | 2.46 | 0.89 |  | 2.07 | 0.988 |  | 4.234 | ****** |
| 2.12 | The gains are higher than the losses | 2.49 | 0.99 |  | 2.18 | 1.093 |  | 3.124 | ****** |
| **2** | **Regulation** | **2.35** | **0.63** |  | **2.05** | **0.61** |  | **5.137** | ****** |
|  | **Factors influencing**  **plagiarism** | **SLO** | |  | **GER** | |  | **t-Test** | |
|  |  | ***M*** | ***SD*** |  | ***M*** | ***SD*** |  | ***t*** | ***p*** |
| 3.1 | I run out of time | 3.37 | 1.02 |  | 3.11 | 1.08 |  | 2.585 | ***** |
| 3.2 | I am unable to cope with the workload | 2.88 | 1.03 |  | 2.81 | 1.01 |  | 0.761 |  |
| 3.3 | I do not know how to cite | 2.62 | 1.00 |  | 2.41 | 1.00 |  | 2.165 | ***** |
| 3.4 | I do not know how to find material | 2.49 | 0.96 |  | 2.21 | 0.99 |  | 3.020 | ****** |
| 3.5 | I do not know how to research | 2.45 | 0.92 |  | 2.07 | 0.91 |  | 4.382 | ****** |
| 3.6 | My reading comprehension skills are weak | 1.81 | 0.82 |  | 1.99 | 0.92 |  | -2.098 | ***** |
| 3.7 | My writing skills are weak | 2.25 | 0.96 |  | 2.14 | 0.96 |  | 1.254 |  |
| 3.8 | I sometimes have difficulty to express my own ideas | 2.64 | 1.04 |  | 2.72 | 1.12 |  | -0.816 |  |
| **3** | **Academic skills** | **2.56** | **0.67** |  | **2.44** | **0.68** |  | **1.939** |  |
|  | **Factors influencing**  **plagiarism** | **SLO** | |  | **GER** | |  | **t-Test** | |
|  |  | ***M*** | ***SD*** |  | ***M*** | ***SD*** |  | ***t*** | ***p*** |
| 4.1 | The tasks are too difficult | 2.91 | 0.92 |  | 2.71 | 0.94 |  | 2.295 | ***** |
| 4.2 | Poor explanation - bad teaching | 2.99 | 1.02 |  | 2.80 | 1.03 |  | 1.970 | ***** |
| 4.3 | Too many assignments in a short time | 3.31 | 0.96 |  | 3.24 | 1.11 |  | 0.751 |  |
| 4.4 | Plagiarism is not explained | 2.75 | 1.15 |  | 2.23 | 1.02 |  | 4.986 | ****** |
| 4.5 | I am not satisfied with course contents | 2.98 | 1.00 |  | 2.58 | 0.93 |  | 4.357 | ****** |
| 4.6 | Teachers do not care | 2.66 | 0.89 |  | 2.24 | 1.05 |  | 4.616 | ****** |
| 4.7 | Teachers do not read students' assignments | 2.51 | 0.90 |  | 2.07 | 1.00 |  | 4.887 | ****** |
| **4** | **Teaching factors** | **2.87** | **0.68** |  | **2.56** | **0.72** |  | **4.827** | ****** |
|  | **Factors influencing**  **plagiarism** | **SLO** | |  | **GER** | |  | **t-Test** | |
|  |  | ***M*** | ***SD*** |  | ***M*** | ***SD*** |  | ***t*** | ***p*** |
| 5.1 | Family pressure | 1.99 | 0.89 |  | 2.16 | 1.14 |  | -1.810 |  |
| 5.2 | Peers pressure | 2.03 | 0.87 |  | 2.29 | 1.03 |  | -3.022 | ****** |
| 5.3 | Under stress | 2.79 | 1.20 |  | 3.08 | 1.18 |  | -2.553 | ***** |
| 5.4 | Faculty pressure | 2.65 | 1.13 |  | 2.71 | 1.13 |  | -0.501 |  |
| 5.5 | Money pressure | 2.35 | 1.14 |  | 2.59 | 1.24 |  | -2.081 | ***** |
| 5.6 | Afraid to fail | 2.78 | 1.16 |  | 3.38 | 1.25 |  | -5.283 | ****** |
| 5.7 | Job pressure | 2.33 | 1.14 |  | 2.81 | 1.25 |  | -4.277 | ****** |
| **5** | **Pressure** | **2.42** | **0.86** |  | **2.71** | **0.91** |  | **-3.522** | ****** |
|  | **Factors influencing**  **plagiarism** | **SLO** | |  | **GER** | |  | **t-Test** | |
|  |  | ***M*** | ***SD*** |  | ***M*** | ***SD*** |  | ***t*** | ***p*** |
| 6.1 | I do not want to look stupid by peers | 2.46 | 1.09 |  | 2.65 | 1.11 |  | -1.846 |  |
| 6.2 | I do not want to look stupid by professor | 2.51 | 1.11 |  | 2.79 | 1.18 |  | -2.525 | ****** |
| 6.3 | I do not want to embarrass my family | 2.40 | 1.13 |  | 2.79 | 1.30 |  | -3.417 | ****** |
| 6.4 | I do not want to embarrass my self | 2.50 | 1.19 |  | 2.90 | 1.30 |  | -3.419 | ****** |
| 6.5 | I focus on how my competences will be judged relative to others | 2.36 | 0.99 |  | 2.78 | 1.05 |  | -4.360 | ****** |
| 6.6 | I am focused on learning according to self-set standards | 2.98 | 1.11 |  | 2.89 | 1.09 |  | 0.820 |  |
| 6.7 | I fear to ask for help | 2.29 | 0.99 |  | 2.26 | 1.04 |  | 0.293 |  |
| 6.8 | My fear to perform poorly motivates me to plagiarize | 2.22 | 0.96 |  | 2.43 | 1.08 |  | -2.240 | ***** |
| 6.9 | Assigned academic work will not help me personally/professionally | 2.20 | 1.07 |  | 2.49 | 1.06 |  | -2.882 | ****** |
| **6** | **Pride** | **2.43** | **0.84** |  | **2.67** | **0.80** |  | **-3.032** | ****** |
|  | **Factors influencing**  **plagiarism** | **SLO** | |  | **GER** | |  | **t-Test** | |
|  |  | ***M*** | ***SD*** |  | ***M*** | ***SD*** |  | ***t*** | ***p*** |
| 7.1 | I do not want to work hard | 2.59 | 1.14 |  | 2.33 | 1.19 |  | 2.366 | ***** |
| 7.2 | I do not want to learn anything, just pass | 2.09 | 0.97 |  | 2.17 | 1.18 |  | -0.778 |  |
| 7.3 | My work is not good enough | 2.18 | 0.88 |  | 2.46 | 1.05 |  | -3.059 | ****** |
| 7.4 | It is easier to plagiarize than to work | 2.73 | 1.16 |  | 2.81 | 1.35 |  | -0.674 |  |
| 7.5 | To get better-higher mark (score) | 2.76 | 1.10 |  | 2.94 | 1.24 |  | -1.592 |  |
| **7** | **Other factors** | **2.47** | **0.82** |  | **2.54** | **0.94** |  | **-0.836** |  |

*Note.* **p* < .05. ***p* < .01
