# Supplementary material for: Factors influencing plagiarism in higher education: A comparison of German and Slovene students
Source: PLoS One. 2018 Aug 10;13(8):e0202252. doi: 10.1371/journal.pone.0202252 (PMC6086479; doi:10.1371/journal.pone.0202252)
Supplement: S3 Table — (DOCX) [file pone.0202252.s003.docx]

**S3 Table. Descriptive statistics for items referring to the factors influencing plagiarism, by gender and results of the t-Test (SLO).**

| **Factors influencing plagiarism** | **Male** | |  | **Female** | |  | **t-Test** | |
| --- | --- | --- | --- | --- | --- | --- | --- | --- |
|  | ***M*** | ***SD*** |  | ***M*** | ***SD*** |  | ***t*** | ***p (1-sided)*** |
| 2.6 | 2.26 | 0.97 |  | 1.98 | 0.84 |  | 2.157 | *** |
| 2.8 | 2.33 | 1.15 |  | 1.93 | 0.89 |  | 2.683 | *** |
| 2.12 | 2.63 | 1.00 |  | 2.34 | 0.97 |  | 2.001 | *** |
| 3.8 | 2.48 | 0.99 |  | 2.80 | 1.07 |  | -2.138 | *** |
| 4.4 | 2.92 | 1.15 |  | 2.56 | 1.13 |  | 2.162 | *** |
| 4.6 | 2.82 | 0.83 |  | 2.49 | 0.92 |  | 2.524 | **** |
| 4.7 | 2.72 | 0.88 |  | 2.29 | 0.87 |  | 3.339 | **** |
| 5.2 | 2.14 | 0.84 |  | 1.90 | 0.89 |  | 1.920 | *** |
| 5.4 | 2.79 | 1.05 |  | 2.51 | 1.21 |  | 1.670 | *** |
| 6.3 | 2.55 | 1.18 |  | 2.24 | 1.06 |  | 1.914 | *** |
| 6.5 | 2.56 | 1.04 |  | 2.14 | 0.90 |  | 2.992 | **** |
| 6.7 | 2.46 | 0.99 |  | 2.11 | 0.96 |  | 2.522 | **** |
| 6.9 | 2.42 | 1.09 |  | 1.97 | 0.99 |  | 2.977 | **** |
| 7.2 | 2.21 | 1.01 |  | 1.96 | 0.91 |  | 1.827 | *** |

*Note.* **p* < .05. ***p* < .01
