# Supplementary material for: Factors influencing plagiarism in higher education: A comparison of German and Slovene students
Source: PLoS One. 2018 Aug 10;13(8):e0202252. doi: 10.1371/journal.pone.0202252 (PMC6086479; doi:10.1371/journal.pone.0202252)
Supplement: S4 Table — (DOCX) [file pone.0202252.s004.docx]

**S4 Table. Descriptive statistics for items referring to the factors influencing plagiarism, by gender and results of the t-Test (GER).**

| **Factors influencing plagiarism** | **Male** | |  | **Female** | |  | **t-Test** | |
| --- | --- | --- | --- | --- | --- | --- | --- | --- |
|  | ***M*** | ***SD*** |  | ***M*** | ***SD*** |  | ***t*** | ***p (1-sided)*** |
| 2.8 | 2.20 | 1.05 |  | 1.94 | 1.00 |  | 2.067 | *** |
| 2.10 | 1.96 | 1.11 |  | 1.70 | 0.92 |  | 2.097 | *** |
| 2.12 | 2.36 | 1.11 |  | 2.02 | 1.06 |  | 2.524 | **** |
| 3.7 | 2.25 | 0.97 |  | 2.04 | 0.94 |  | 1.768 | *** |
| 4.1 | 2.58 | 0.98 |  | 2.77 | 0.91 |  | -1.672 | *** |
| 4.7 | 2.23 | 1.00 |  | 1.94 | 0.96 |  | 2.363 | **** |
| 7.1 | 2.50 | 1.24 |  | 2.18 | 1.12 |  | 2.168 | *** |
| 7.4 | 3.01 | 1.38 |  | 2.68 | 1.31 |  | 2.001 | *** |
| 7.5 | 3.13 | 1.18 |  | 2.80 | 1.24 |  | 2.112 | *** |

Note. **p* < .05. ***p* < .01
