# Supplementary material for: Factors influencing plagiarism in higher education: A comparison of German and Slovene students
Source: PLoS One. 2018 Aug 10;13(8):e0202252. doi: 10.1371/journal.pone.0202252 (PMC6086479; doi:10.1371/journal.pone.0202252)
Supplement: S5 Table — (DOCX) [file pone.0202252.s005.docx]

**S5 Table. Descriptive statistics for items referring to the factors influencing plagiarism, by area of study and results of the One-Way ANOVA (SLO).**

| **Factors influencing plagiarism** | **Area of study** | | | | | | | |  | **ANOVA** | |
| --- | --- | --- | --- | --- | --- | --- | --- | --- | --- | --- | --- |
|  | **Technical** | |  | **Social** | |  | **Natural** | |  |  |  |
|  | ***M*** | ***SD*** |  | ***M*** | ***SD*** |  | ***M*** | ***SD*** |  | ***F*** | ***p*** |
| 1.2 | 2.61 | 1.08 |  | 2.17 | 1.04 |  | 2.47 | 0.90 |  | 3.562 | *** |
| 2.4  2.12 | 2.23 | 0.86 |  | 1.87 | 0.89 |  | 2.10 | 0.80 |  | 3.396 | *** |
|  | 2.74 | 1.01 |  | 2.34 | 1.01 |  | 2.47 | 0.78 |  | 3.329 | *** |
| 4.2 | 3.23 | 1.06 |  | 2.80 | 0.93 |  | 3.20 | 1.06 |  | 4.246 | *** |
| 4.4 | 3.00 | 1.19 |  | 2.48 | 1.06 |  | 3.00 | 1.17 |  | 5.035 | **** |
| 4.5 | 3.26 | 1.00 |  | 2.77 | 0.98 |  | 3.07 | 0.94 |  | 4.804 | **** |
| 4.6 | 2.97 | 0.88 |  | 2.44 | 0.81 |  | 2.72 | 0.92 |  | 7.485 | **** |
| 4.7 | 2.86 | 0.93 |  | 2.30 | 0.82 |  | 2.43 | 0.86 |  | 8.365 | **** |
| 5.4  5.6 | 2.97 | 1.11 |  | 2.51 | 1.12 |  | 2.43 | 1.14 |  | 4.036 | *** |
|  | 3.20 | 1.15 |  | 2.61 | 1.12 |  | 2.47 | 1.08 |  | 6.693 | **** |
| 6.5 | 2.67 | 1.07 |  | 2.18 | 0.85 |  | 2.27 | 1.08 |  | 5.003 | **** |
| 6.6 | 3.29 | 1.02 |  | 2.83 | 1.13 |  | 2.77 | 1.14 |  | 4.091 | *** |
| 6.7 | 2.59 | 1.04 |  | 2.14 | 0.94 |  | 2.17 | 0.91 |  | 4.512 | *** |

*Note.* **p* < .05. ***p* < .01
