# Supplementary material for: Factors influencing plagiarism in higher education: A comparison of German and Slovene students
Source: PLoS One. 2018 Aug 10;13(8):e0202252. doi: 10.1371/journal.pone.0202252 (PMC6086479; doi:10.1371/journal.pone.0202252)
Supplement: S6 Table — (DOCX) [file pone.0202252.s006.docx]

**S6 Table. Descriptive statistics for items referring to the factors influencing plagiarism, by study area and results of the t-Test (GER).**

|  | **Area of study** | | | | |  |  | |
| --- | --- | --- | --- | --- | --- | --- | --- | --- |
| **Factors influencing plagiarism** | **Technical** | |  | **Social** | |  | **t-Test** | |
|  | ***M*** | ***SD*** |  | ***M*** | ***SD*** |  | ***t*** | ***p (1-sided)*** |
| 2.3 | 1.97 | 0.90 |  | 1.76 | 0.84 |  | 1.872 | *** |
| 2.4 | 1.93 | 0.99 |  | 1.64 | 0.91 |  | 2.374 | **** |
| 2.8 | 2.33 | 1.04 |  | 1.86 | 0.96 |  | 3.536 | **** |
| 2.9 | 2.23 | 1.05 |  | 1.90 | 1.06 |  | 2.342 | *** |
| 2.10 | 2.08 | 1.07 |  | 1.57 | 0.85 |  | 3.860 | **** |
| 2.11 | 2.41 | 0.98 |  | 1.83 | 0.90 |  | 4.676 | **** |
| 2.12 | 2.34 | 1.02 |  | 2.01 | 1.10 |  | 2.323 | *** |
| 3.2 | 2.92 | 0.89 |  | 2.69 | 1.03 |  | 1,866 | *** |
| 4.4 | 2.57 | 1.03 |  | 2.00 | 0.96 |  | 4.424 | **** |
| 4.5 | 2.77 | 0.93 |  | 2.43 | 0.88 |  | 2.923 | **** |
| 4.6 | 2.38 | 1.04 |  | 2.14 | 1.05 |  | 1.739 | *** |
| 4.7 | 2.40 | 1.01 |  | 1.88 | 0.96 |  | 4.019 | **** |
| 6.7 | 2.53 | 1.07 |  | 2.12 | 0.99 |  | 3.041 | **** |
| 6.8 | 2.62 | 1.04 |  | 2.32 | 1.07 |  | 2.170 | *** |
| 6.9 | 2.75 | 0.95 |  | 2.33 | 1.10 |  | 3.155 | **** |
| 7.1 | 2.59 | 1.22 |  | 2.17 | 1.14 |  | 2.760 | **** |
| 7.2 | 2.51 | 1.17 |  | 1.99 | 1.15 |  | 3.377 | **** |
| 7.3 | 2.65 | 1.06 |  | 2.37 | 1.04 |  | 2.014 | *** |
| 7.4 | 3.05 | 1.30 |  | 2.63 | 1.34 |  | 2.474 | **** |
| 7.5 | 3.27 | 1.18 |  | 2.72 | 1.21 |  | 3.540 | **** |

*Note.* **p* < .05. ***p* < .01
