# Supplementary material for: Factors influencing plagiarism in higher education: A comparison of German and Slovene students
Source: PLoS One. 2018 Aug 10;13(8):e0202252. doi: 10.1371/journal.pone.0202252 (PMC6086479; doi:10.1371/journal.pone.0202252)
Supplement: S7 Table — (DOCX) [file pone.0202252.s007.docx]

**S7 Table. Descriptive statistics for items referring to the factors influencing plagiarism, by motivation and results of the t-Test (SLO).**

|  | **Motivation for study** | | | | |  |  | |
| --- | --- | --- | --- | --- | --- | --- | --- | --- |
| **Factors influencing plagiarism** | **Lower** | |  | **Higher** | |  | **t-Test** | |
|  | ***M*** | ***SD*** |  | ***M*** | ***SD*** |  | ***t*** | ***p (1-sided)*** |
| 2.1 | 2.27 | 0.85 |  | 2.55 | 0.88 |  | -2.043 | *** |
| 3.1 | 3.63 | 1.11 |  | 3.24 | 0.95 |  | 2.429 | **** |
| 4.6 | 2.86 | 0.89 |  | 2.58 | 0.88 |  | 2.048 | *** |
| 5.4 | 2.97 | 1.16 |  | 2.50 | 1.09 |  | 2.663 | **** |
| 6.6 | 2.76 | 1.04 |  | 3.08 | 1.13 |  | -1.858 | *** |
| 6.9 | 2.49 | 1.17 |  | 2.07 | 1.00 |  | 2.556 | **** |
| 7.1 | 2.90 | 1.27 |  | 2.46 | 1.05 |  | 2.496 | **** |
| 7.2 | 2.66 | 1.11 |  | 1.83 | 0.79 |  | 5.188 | **** |
| 7.3 | 2.44 | 0.97 |  | 2.07 | 0.82 |  | 2.566 | **** |

*Note.* **p* < .05. ***p* < .01
