# Supplementary material for: Factors influencing plagiarism in higher education: A comparison of German and Slovene students
Source: PLoS One. 2018 Aug 10;13(8):e0202252. doi: 10.1371/journal.pone.0202252 (PMC6086479; doi:10.1371/journal.pone.0202252)
Supplement: S8 Table — (DOCX) [file pone.0202252.s008.docx]

**S8 Table. Descriptive statistics for items referring to the factors influencing plagiarism, by motivation and results of the t-Test (GER).**

|  | **Motivation for study** | | | | |  |  | |
| --- | --- | --- | --- | --- | --- | --- | --- | --- |
| **Factors influencing plagiarism** | **Lower** | |  | **Higher** | |  | **t-Test** | |
|  | ***M*** | ***SD*** |  | ***M*** | ***SD*** |  | ***t*** | ***p (1-sided)*** |
| 2.7 | 2.44 | 0.95 |  | 2.31 | 0.93 |  | 1.959 | * |
| 2.8 | 2.33 | 1.04 |  | 1.86 | 0.96 |  | 2.832 | **** |
| 2.9 | 2.23 | 1.05 |  | 1.90 | 1.06 |  | 2.459 | **** |
| 2.10 | 2.08 | 1.07 |  | 1.57 | 0.85 |  | 2.128 | *** |
| 2.12 | 2.34 | 1.02 |  | 2.01 | 1.10 |  | 2.281 | *** |
| 4.1 | 2.71 | 0.89 |  | 2.65 | 0.95 |  | 2.559 | **** |
| 4.2 | 2.84 | 0.98 |  | 2.72 | 1.06 |  | 2.463 | **** |
| 4.5 | 2.77 | 0.93 |  | 2.43 | 0.88 |  | 2.664 | **** |
| 4.6 | 2.38 | 1.04 |  | 2.14 | 1.05 |  | 1.840 | *** |
| 6.9 | 2.75 | 0.95 |  | 2.33 | 1.10 |  | 1.667 | *** |
| 7.1 | 2.59 | 1.22 |  | 2.17 | 1.14 |  | 3.031 | **** |
| 7.2 | 2.51 | 1.17 |  | 1.99 | 1.15 |  | 3.286 | **** |
| 7.3 | 2.65 | 1.06 |  | 2.37 | 1.04 |  | 2.716 | **** |
| 7.4 | 3.05 | 1.30 |  | 2.63 | 1.34 |  | 2.209 | *** |
| 7.5 | 3.27 | 1.18 |  | 2.72 | 1.21 |  | 2.763 | **** |

*Note.* **p* < .05. ***p* < .01
